# pH-responsive and hyaluronic acid-functionalized metal–organic frameworks for therapy of osteoarthritis

**DOI:** 10.1186/s12951-020-00694-3

**Published:** 2020-09-29

**Authors:** Feng Xiong, Zainen Qin, Haimin Chen, Qiumei Lan, Zetao Wang, Nihan Lan, Yuan Yang, Li Zheng, Jinmin Zhao, Dan Kai

**Affiliations:** 1 Guangxi Engineering Center in Biomedical Material for Tissue and Organ Regeneration, The First Affiliated Hospital of Guangxi Medical University, Nanning, 530021 China; 2 Guangxi Collaborative Innovation Center for Biomedicine, The First Affiliated Hospital of Guangxi Medical University, Nanning, 530021 China; 3 Department of Orthopaedics Trauma and Hand Surgery, The First Affiliated Hospital of Guangxi Medical University, Nanning, 530021 China; 4 Orthopaedics, Langdong Hospital of Guangxi Medical University, The First Affiliated Hospital of Guangxi Medical University, Nanning, 530021 China; 5 Life Sciences Institute, Guangxi Medical University, Nanning, 530021 China; 6 Guangxi Key Laboratory of Regenerative Medicine, Life Sciences Institute, The First Affiliated Hospital of Guangxi Medical University, Nanning, 530021 China; 7 Institute of Materials Research and Engineering (IMRE), A*STAR, 2 Fusionopolis Way, #08-03, Innovis, 138634 Singapore

**Keywords:** Osteoarthritis, pH-responsive, Metal–organic frameworks, Protocatechuic acid

## Abstract

Drug therapy of osteoarthritis (OA) is limited by the short retention and lacking of stimulus-responsiveness after intra-articular (IA) injection. The weak acid microenvironment in joint provides a potential trigger for controlled drug release systems in the treatment of OA. Herein, we developed an pH-responsive metal − organic frameworks (MOFs) system modified by hyaluronic acid (HA) and loaded with an anti-inflammatory protocatechuic acid (PCA), designated as MOF@HA@PCA, for the therapy of OA. Results demonstrated that MOF@HA@PCA could smartly respond to acidic conditions in OA microenvironment and gradually release PCA, which could remarkably reduce synovial inflammation in both IL-1β induced chondrocytes and the OA joints. MOF@HA@PCA also down-regulated the expression of inflammatory markers of OA and promoted the expression of cartilage-specific makers. This work may provide a new insight for the design of efficient nanoprobes for precision theranostics of OA
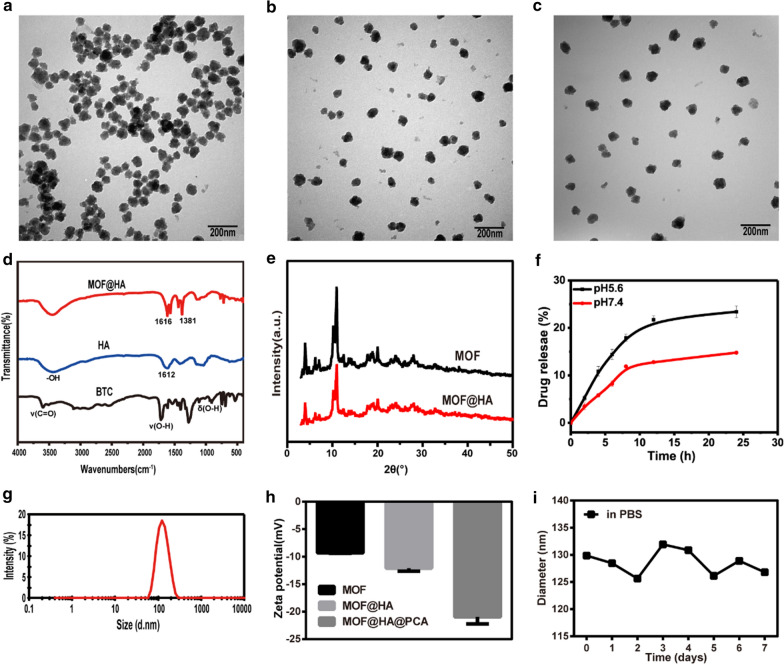
.

## Introduction

Osteoarthritis (OA) is a chronic disease that characterized by progressive degeneration of cartilage, inflammation of synovial membrane and abnormal reduction of lubrication in joint [1, 2]. When OA occurs, it may cause pains, damage and dysfunctions in joint, or even millions of people with serious disabilities at the end-stage [3]. Although intra-articular (IA) drug injection is an attractive way to improve the efficacy of OA, free drugs are cleared from the joint cavity rapidly, resulting in reduced drug bioavailability and increased complications [4, 5].

Recently, nanoparticle-based drug delivery systems, including metal nanoparticles [6], liposomes [7, 8] and conventional micelles [8, 9], have been introduced into OA therapeutics to prolong residence time of small drug molecules in joint cavity. However, these systems are always loaded with limited amounts of drugs and are not smart stimulus-responsive, which can hardly meet the requirement for OA therapy. The reason may be due to the instability in OA synovial fluid and the lacking of controlled drug release kinetics according to the severity of OA. The previous studies showed that inflamed-joint is marked by weakly acidic environment (may down to 6.0) during the process of cartilage degradation [10–13], providing a suitable trigger to drug control release. In order to stably deliver drug into OA-affected site without undesired drug early leakage and increase treatment effect by controlled release the drug to inflamed chondrocytes, it is necessary to develop a smart pH-stimuli-responsive drug control release system for precisely treating of OA.

Metal − organic frameworks (MOFs), a new class of developed porous materials with abundant metal sites [14–16], have been utilized in various fields, such as drug delivery systems [17–19], molecular imaging [18, 19] and biological sensing [20, 21]. Among the MOFs, MIL-100(Fe) is widely used as a drug carrier due to their notable properties, such as high drug loading capacity (due to its dense pores and large pore size), excellent biocompatibility and pH-responsive property [22–24]. The bottleneck problem for MIL-100(Fe) is poor water stability [16, 25], which barriers its application to therapy of OA. Modification with hydrophilic agent hyaluronic acid (HA) can improve the dispersibility and hydrophily of MIL-100(Fe) [26]. Moreover, in OA joints, the HA in synovial fluid and cartilage surface is degraded by inflammation factors, causing lower viscosity and further disease progression [27–29]. The artificially adding HA is particularly known for its potential properties, including reducing inflammation [30, 31], lubricating cartilage and protecting chondrocytes from free-radical damage [3, 32], which can relieve pain and protect joints to promote cartilage regeneration [2, 30–33].

In this study, we report an advanced pH-responsive system modified by the natural cartilage lubricant, HA [34], aiming to develop a smart bioengineered drug control release carrier (designated as MOF@HA@PCA) for OA therapy. The MOF@HA-NPs were used to deliver an anti-inflammatory agent protocatechuic acid (PCA), for controlled release in response to acidic environment of OA by using the pH-responsive property of MOF@HA. Furthermore, PCA is a polyphenolic compound isolated from plants [35] that has potent anti-inflammatory properties since it can significantly down-regulate the indicators of inflammatory factors including inducible nitric oxide synthase (iNOS), cyclooxygenase-2 (COX2) and metalloproteinase with thrombospondin motifs (ADAMTSs) [36, 37]. It is well known that the reduced production of these inflammatory mediators can inhibit cells damage in OA-affected site to prevent tissue injury in the progress of OA [38–40]. This system may provide therapeutic strategy for OA.

## Materials and methods

### Synthesis of MIL-100(Fe) NPs (MOF NPs) and MOF@HA NPs

MIL-100(Fe) NPs were synthesized by the method reported in the literature [41] and subsequently conjugated to HA to form a multifunctional nanoplatform MOF@HA. In a typical procedure, firstly, 80 mL of 7.5 mM FeCl_3_ solution (in methanol) (Sigma-Aldrich, USA) and 80 mL of 7.5 mM 1, 3, 5-benzenetricarboxylic acid (BTC) solution (in methanol) (Sigma-Aldrich, USA) were mixed under strong stirring for about 10 min. Afterward, the mixture was transferred into a 55 °C oil bath and reacted for 4 h. The reaction product was collected by 8000 rpm centrifugation and washed 2 times with ethanol and deionized water. Finally, the product was stored after freeze-drying. The MOF@HA NPs could be obtained by mixing MOF NPs with HA (Sigma-Aldrich, USA) at weight ratio of 2:1 for 24 h.

### MOF stability and degradation study

MOF NPs were dissolved in deionized water with concentration of 0.1 mg/mL, following by dispersing in PBS solution. After incubation for different time (0, 1, 2, 3, 4, 5, 6 and 7 d), the diameters of NPs were monitored by a dynamic light scattering (DLS) (Malvern, UK).

The degradation of MOF NPs were quantitative by measurement the release of Fe^3+^ triggered by an acidic environment. The MOF NPs were resuspended in PBS of different pH (pH 7.4 and 5.6) and reacted with the system of ophenanthroline (see the Supporting information in detail) for different time (1, 2 and 3 d). The absorbance was then detected with a microplate reader at 510 nm and the Fe^3+^ released were calculated quantitatively.

### Drug loading

The MOF@HA@PCA NPs could be obtained by mixing PCA (Aladdin, China) with MOF@HA NPs at weight ratio of 2:1 solutions, followed by about 24 h of shaking in the dark. The mixtures were centrifuged under 8000 rpm, then the precipitations were washed in aqueous solutions for three times to remove the unloaded free PCA. The MOF@HA NPs were obtained after lyophilization.

The unloaded free PCA in the solution was analyzed by High Performance Liquid Chromatography (HPLC) (Shimadzu, Japan) with the analytical column Red Classical AQ-C18 column (250×4.6 mm) quantified at the wavelength of 360 nm. The mobile phase includes methanol and water (contain 1% glacial acetic acid) in a ratio of 30:70 (v/v). All chromatography was carried out under the flow rate of 1.0 mL/min at room temperature. 20 μL supernatants were loaded into the HPLC column for all the analyses. The drug loading capacity (LC, %) and drug encapsulation efficiency (EE, %) were calculated by the following formula (1), (2):

1 $${\text{LC }} \left( {\% } \right) = \frac{{\text{weight of drug feeding } - \text{ weight of drug unloaded}}}{{\text{weight of particle}}} \times 100\%$$

2 $${\text{EE }} \left( {\% } \right) = \frac{{\text{weight of drug feeding } - \text{ weight of drug unloaded}}}{{\text{weight of drug feeding}}} \times 100\%$$

### Characterization

The chemical structure of MOF, MOF@HA, and MOF@HA@PCA NPs were obtained by Fourier transform infrared spectroscopy (FT − IR) (Perkin Elmer, USA). Quantify the amount of HA on MIL-100(Fe) NPs by electronic balance (METTLER TOLEDO, USA) (see the Supporting information in detail). X-ray diffraction (XRD) (Rigaku, Japan) was utilized to characterize the crystalline structure. The size and morphology of nanoparticles were obtained by transmission electron microscopy (TEM) (Bruker, Germany). The size statistics of the MOF NPs and the zeta potential of MOF, MOF@HA, and MOF@HA@PCA NPs were measured by a dynamic light scattering (DLS) (Malvern, UK).

### Release of PCA from MOF@HA@PCA

The PCA release experiments were performed at pH 5.6 and 7.4, respectively. 10 mg of MOF@HA@PCA NPs was immersed in 10 mL PBS of two different pH values. The PBS was collected and replaced with the same PBS at each time point (0, 2, 4, 6, 8, 12, 24 h). The concentration of PCA in the collected PBS were measured by HPLC. The detection method and parameters of HPLC are performed according to the above process.

### Isolation and culture of chondrocytes

Primary chondrocytes were obtained from 3 day-old Sprague Dawley (SD) rats. The articular cartilage was digested with 0.25% trypsin–EDTA (Solarbio, China) for 0.5 h to remove other tissues. Then, 0.2% collagenase II (Sigma-Aldrich, China) was used for digestion at 37 °C for 4 h. The chondrocytes were collected by centrifugation at 1000 rpm for 5 min and cultured in Dulbecco's Modified Eagle’s Medium–High glucose (DMEM, Gibco, USA) that supplemented with 10% fetal bovine serum (FBS, Hyclone, USA) and 1% penicillin/streptomycin (Solarbio, China). The cells were incubated at 37 C in a humidified atmosphere with 5% CO_2_. The chondrocytes at passage 3 were utilized for subsequent experiments.

### Cell cytotoxicity analysis

The cytotoxicity of MOF, MOF@HA and MOF@HA@PCA was evaluated by MTT assay. Cells were cultured in a 96-well plate at a density of 5000 cells/well. Then, the cells were pre-treated with different concentrations of MOF (0, 5, 10, 20, 30, 40, 50, 60, 70, 80, 90, 100 μg/mL), MOF@HA (0, 5, 10, 20, 30, 40, 50, 60, 70, 80, 90, 100 μg/mL) or MOF@HA@PCA (0, 5, 10, 20, 30, 40, 50, 60, 70, 80, 90, 100 μg/mL) for 1 h, and then induced by 10 ng/mL of IL-1β for 24 h. Then, 20 μL of MTT was added to each well and the chondrocytes were incubated at 37 °C for 4 h. After the MTT medium was removed, dimethyl sulfoxide (DMSO) was added to dissolve purple triphenylmethylamine crystals produced by living cells. The absorbance was measured at 490 nm using a microplate reader (Thermo Fisher Scientific, USA).

### Cell viability assay

The Live/dead cells assay was performed using a live/dead viability assay kit (Invitrogen, USA). The cells were washed by PBS and incubated with the solution containing 2 μM of calcein AM and 4 μM of ethidium homodimer-1 for 30 min at room temperature in the dark. Then the cells were washed with PBS before observed using a fluorescent microscope (Olympus, Japan). As described in the manufacturer's protocol, live cells were stained green and dead cells were stained red.

### Intracellular ROS measurement

Reactive Oxygen Species Assay Kit (Beyotime Biotechnology, China) was used to detect the ROS generation of intracellular induced by MOF. Cells were divided into three groups: (1) Control group: chondrocytes treated with culture medium only; (2) MOF group: chondrocytes treated with 30 mg/mL MOF for 24 h; (3) Rosup group: chondrocytes treated with culture medium only followed by stimulating with 1 μL 50 mg/mL active oxygen positive control reagent (Rosup) for 30 min. Chondrocytes were washed with serum-free medium and loaded fluorogenic probes 2′, 7′-dichlorofluorescein diacetate (DCFH-DA). After incubation with 10 μM of DCFH-DA at 37 °C for 30 min, the cells were washed three times with serum-free medium. Subsequently, determination of relative fluorescent intensity by using a laser scanning confocal microscopy (Nikon A1, Japan) and statistics of ROS levels by using image J, respectively.

### Treatment of inflamed-chondrocytes induced by IL-1β

Chondrocytes were divided into four groups: (1) Control group: chondrocytes treated with culture medium only; (2) IL-1β group: chondrocytes stimulated with 10 ng/mL IL-1β (Gibco, USA) for 24 h; (3) PCA group: chondrocytes pre-incubated with 6 μg/mL PCA for 1 h followed by stimulating with 10 ng/mL IL-1β for 24 h; (4) MOF@HA@PCA group: chondrocytes pre-incubated with MOF@HA@PCA (containing 6 μg/mL of PCA) for 1 h followed by stimulating with 10 ng/mL IL-1β for 24 h.

### Quantitative real-time polymerase chain reaction (qRT-PCR) analysis

Total RNA was isolated from chondrocytes using an RNA isolation kit (Margen, China) and the qRT-PCR was performed according to the study previously reported [42]. The primer sequences used for qRT-PCR were presented in Table 1.

**Table 1:** The primer sequences used for qRT-PCR

Gene	Forward primer	Reverse primer
GAPDH	TCCAGTATGACTCTACCCACG	CACGACATACTCAGCACCAG
*Acan*	GACAAGGACGAGTTCCCTGG	CTCCGGGGATGTGGCATAAA
*Col2a1*	GTCCTACAATGTCAGGGCCA	ACCCCTCTCTCCCTTGTCAC
*Adamts5*	GCCAAATGGTGTGTCTGACC	CGCAAGAGCGAGAACACTGA
*COX2*	GATGACGAGCGACTGTTCCA	CAATGTTGAAGGTGTCCGGC
*Il6*	ACAAGTCCGGAGAGGAGACT	ACAGTGCATCATCGCTGTTC
*iNos*	GGTGAGGGGACTGGACTTTTAG	TCTCCGTGGGGCTTGTAGTT
*Mmp1*	TGGACCTGAATATGGACTTGCT	GCTGGATGGGATTTGGGGAA
*Mmp3*	GGCTGTGTGCTCATCCTACC	TGGAAAGGTACTGAAGCCACC
*Mmp13*	GGACAAAGACTATCCCCGCC	GGCATGACTCTCACAATGCG

### Safranin O and immunofluorescence staining in vitro

For in vitro study, the cells in all groups were fixed with 95% alcohol for 30 min. After washing with PBS, the cells were stained with safranin O (Sigma, USA) for 10 min and then washed with PBS to remove residual dye.

The cells were washed with PBS and incubated 3% (v/v) hydrogen peroxide H_2_O_2_ to block endogenous peroxidase activity for 15 min at room temperature. After blocking with normal goat serum for 20 min at room temperature, primary MMP-13 antibodies were incubated (1:200 dilution, Abcam, USA) at 4 °C overnight. After incubation with fluorescent secondary antibody at room temperature for 1 h, the nucleus were stained with 4′, 6-diamidino-2-phenylindole (DAPI, Solarbio, China) for 5 min. Images were acquired on a microscope (Olympus, Japan).

### Animal procedure

A total of 42 male Sprague Dawley (SD) rats (10-week-old, weight 200 ± 10 g) obtained from Animal Research Committee of Guangxi Medical University (Nanning, China) were used in the animal experiments. All procedures were carried out according to the guide for the care and use of laboratory animals.

The OA model was surgically induced by anterior cruciate ligament transection (ACLT) according to previous reports [43]. After the rats were anesthetized by intraperitoneal injection of pentobarbital sodium (30 mg/kg), 36 rats underwent bilateral ACLT on the knee joints to induce OA. Other 6 rats underwent sham operations (sham group), in which the articular cavity was opened and the intact short anterior cruciate ligament was sutured. After surgery for one month, the rats were randomly sorted into three groups: NS group, IA injections of 0.5 mL saline; PCA group, IA injections of 0.5 mL saline with PCA (6 μg/mL); MOF@HA@PCA group, IA injections of 0.5 mL saline with MOF@HA@PCA (containing 6 μg/mL of PCA). IA injections were performed once a week. The rats in these groups were sacrificed for analysis at 4 and 8 weeks after therapy.

### Macroscopic observation

After 4 and 8 weeks of treatment, the rats were sacrificed by intraperitoneal injection of excess pentobarbital. The knee joints were collected and changes of cartilage were assessed and scored by three independent observers who were blinded to the treatment groups. The observers evaluated the depth of lesions of articular cartilage on a scale of 0–4 as described by Pelletier et.al [44]. (0 = normal-appearing surface, 1 = minimal fibrillation or a slight yellow discoloration of the surface, 2 = erosion extending into the superficial or middle layers only, 3 = erosion extending into the deep layers and 4 = erosion extending to the subchondral bone).

### Histological staining in vivo

The rats were sacrificed and the knee joints were collected and fixed in 4% paraformaldehyde for 48 h, then decalcified in 10% ethylenediaminetetraacetate (EDTA) for 4 weeks. After serial dehydration, the joints were embedded in paraffin and sagittally sectioned at 3 μm thickness. The sections were de-waxed and stained with hematoxylin–eosin (HE, Jian Cheng Biotech, China) and safranin O/fast green (Solarbio, Beijing, China). Then, three independent observers graded the sections based on the scoring criteria reported by Osteoarthritis Research Society International (OARSI) [45].

The immunohistochemical staining was used to analyze the secretion of MMP-13. The dewaxed sections in the different groups were washed with PBS and exposed to 3% (v/v) hydrogen peroxide H_2_O_2_ to block endogenous peroxidase activity for 15 min at room temperature. After blocking with normal goat serum for 20 min at room temperature, primary MMP-13 antibodies were added (1:200 dilution, Abcam, USA) and incubated at 4 °C overnight. The sections were incubated with secondary antibody for 15 min and then added with biotin-labeled horse radish peroxidase for 15 min. A 3, 3′-diaminobenzidine tetrahydrochloride (DAB) kit and hematoxylin were used for color development and nuclei dye. Tissues sections were observed and photographed with a microscope.

### Statistical analysis

Statistical analyses were performed by SPSS statistics 22.0. All data were expressed as the mean ± standard deviation (SD), and all independent experiments were repeated at least three times. One-way analysis of variance (ANOVA) was used to assess group differences. *P* < 0.05 was considered statistically significant.

## Results

### Characterizations of nanoparticles

**Fig. 1:**
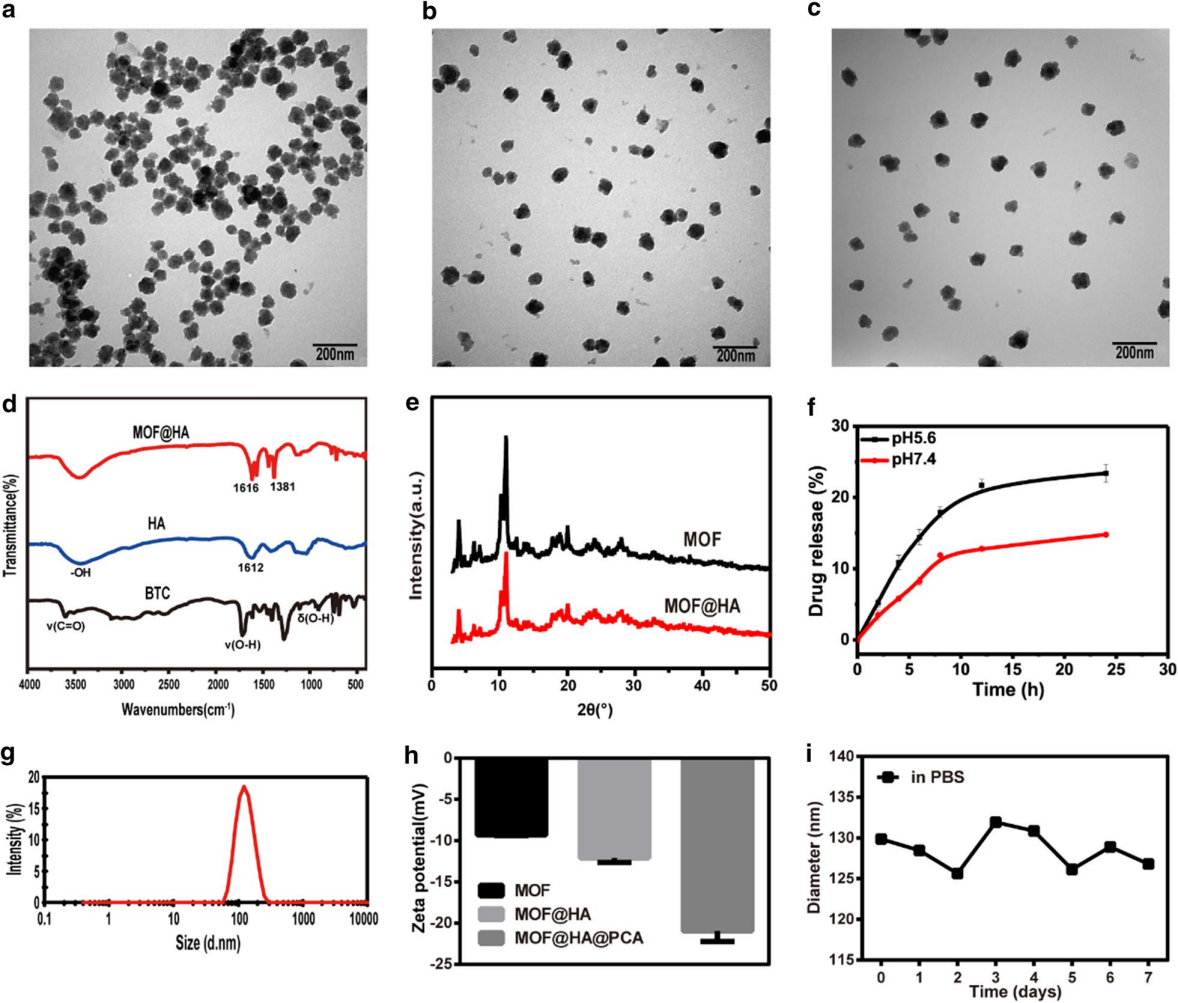
TEM images of **a** MOF, **b** MOF@HA and **c** MOF@HA@PCA. **d** FTIR spectra of BTC, HA, and MOF@HA. **e** XRD spectra of MOF and MOF@HA. **f** Cumulative in vitro drug release profile of PCA in PBS (pH 5.6, 7.4) from MOF@HA@PCA. Data was represented as mean ± SD (n = 3). **g** DLS of the MOF@HA@PCA. **h** Zeta potential of MOF, MOF@HA and MOF@HA@PCA. **i** The stability analysis of MOF. (n = 3, mean ± SD)

TEM images showed that the size of MOF, MOF@HA and MOF@HA@PCA were around 100 nm (Fig. 1a–c). The distance between particles became larger for MOF@HA and MOF@HA@PCA due to the surface modification of HA, indicating their excellent dispersibility and hydrophilicity. FTIR spectroscopy was carried out to study the chemical structure of the products (Fig. 1d). The peaks at 1612 and 3200 − 3600 cm^−1^ originated from the amide band and the − OH group in HA, respectively. The three peaks that appeared at 3300 − 2500, 1720 − 1680, and 950 − 890 cm^−1^ were assigned to the absorption peaks of ν (C = O), ν (O − H), and δ (O − H) in BTC, respectively. Two apparent peaks at 1616 and 1381 cm^−1^, corresponding to the asymmetric and symmetric stretching vibrations of − COO − anions, were found in the MOF@HA NPs spectra. These results further confirmed the formation of MIL-100(Fe) product in our experiment and that HA had been successfully modified with MOF NPs [46–48]. XRD was used to identify the crystal structure and phase composition of the products. Figure 1e presents the XRD pattern of the MIL-100(Fe) NPs before and after coating of HA. All the diffraction peaks in the XRD patterns could be identified as a cubic structure, which is in agreement with previous reports [49, 50]. The conjugated of HA did not change the crystalline structure of MIL-100(Fe) NPs but slightly decreased the reflection intensity. The DLS size measurement in deionized water (Fig. 1g) revealed that the average size of the MOF@ HA@PCA NPs was 123.4 nm. The polydispersity index of MOF@HA@PCA NPs was 0.134, indicating the size distributions of NPs was uniform. The changes of the zeta potential in MOF, MOF@HA, and MOF@HA@PCA NPs were characterized (Fig. 1h). Because of the presence of HA on MOF NPs, the zeta potential of MOF@HA NPs decreased from − 9.3 to − 12.1 mV, then it decreased to − 21 mV after encapsulation of PCA molecules due to the − OH groups on the surface of PCA. The rate of HA on MIL-100(Fe) NPs (HA, %) was 21.6%.

### Drug loading and release in vitro

The drug loading ratio of MOF@HA@PCA NPs was 19.4%. The drug encapsulation efficiency ratio of MOF@HA@PCA NPs was 38.8%. And the results showed the sustained and controlled release of PCA from MOF@HA@PCA NPs at pH 5.6 and 7.4 in vitro, respectively (Fig. 1f). PCA released quickly in the first few hours, and the release rate reached its maximum within 24 h. At pH 7.4, only 14.8% PCA released from MOF@HA@PCA, while the drug release rate obviously accelerated at pH 5.6 (23.4%) at 24 h, implying that the drug release could be due to pH-induced disassembly of the NPs. The protonation of MOF@HA@PCA in acidic condition may lead to their discharge and subsequently can cause higher PCA release in acidic media [51]. The MIL-100(Fe) degraded in PBS at pH 7.4 for a long time, and with the decrease of the pH values of PBS media, the MIL- 100(Fe) exhibits accelerated degradation. This is partially related to degradation of the MIL-100(Fe) structure to release the ferric ions in in acidic PBS media, resulting in the significantly increased anti-inflammatory drugs PCA release [52, 53].

### MOF stability and degradation in vitro

After incubating with MOF in PBS (pH 7.4) for 1, 2 and 3 d, unobvious degradation from MIL-100(Fe) was detected by the method of o-phenanthroline, and the concentrations of Fe^3+^ in supernatants were 1.4, 2 and 3.6 μΜ, respectively. However, when incubated at acidic conditions (pH 5.6), with prolonging incubation times (1, 2 and 3 d), the degradation of MIL-100(Fe) accelerated and the concentrations of Fe^3+^ were 3.7, 4.6 and 5.5 μΜ, respectively (Additional file 1. Fig. S1–S6). Compared to that in the solution at pH 7.4, the Fe^3+^ released in acidic solution increased, indicating MOF NPs display an acid-triggered degradation behavior, and it could be beneficial for MOF NPs to realize pH-sensitive drug release at OA site.

DLS was further utilized to monitor the stability over time. The particle sizes of MOF NPs showed a slight variation in the range of 125-135 nm (Fig. 1i), indicating that MOF exhibited excellent stability within 7 days.

### MOF@HA@PCA promote proliferation of chondrocytes

**Fig. 2:**
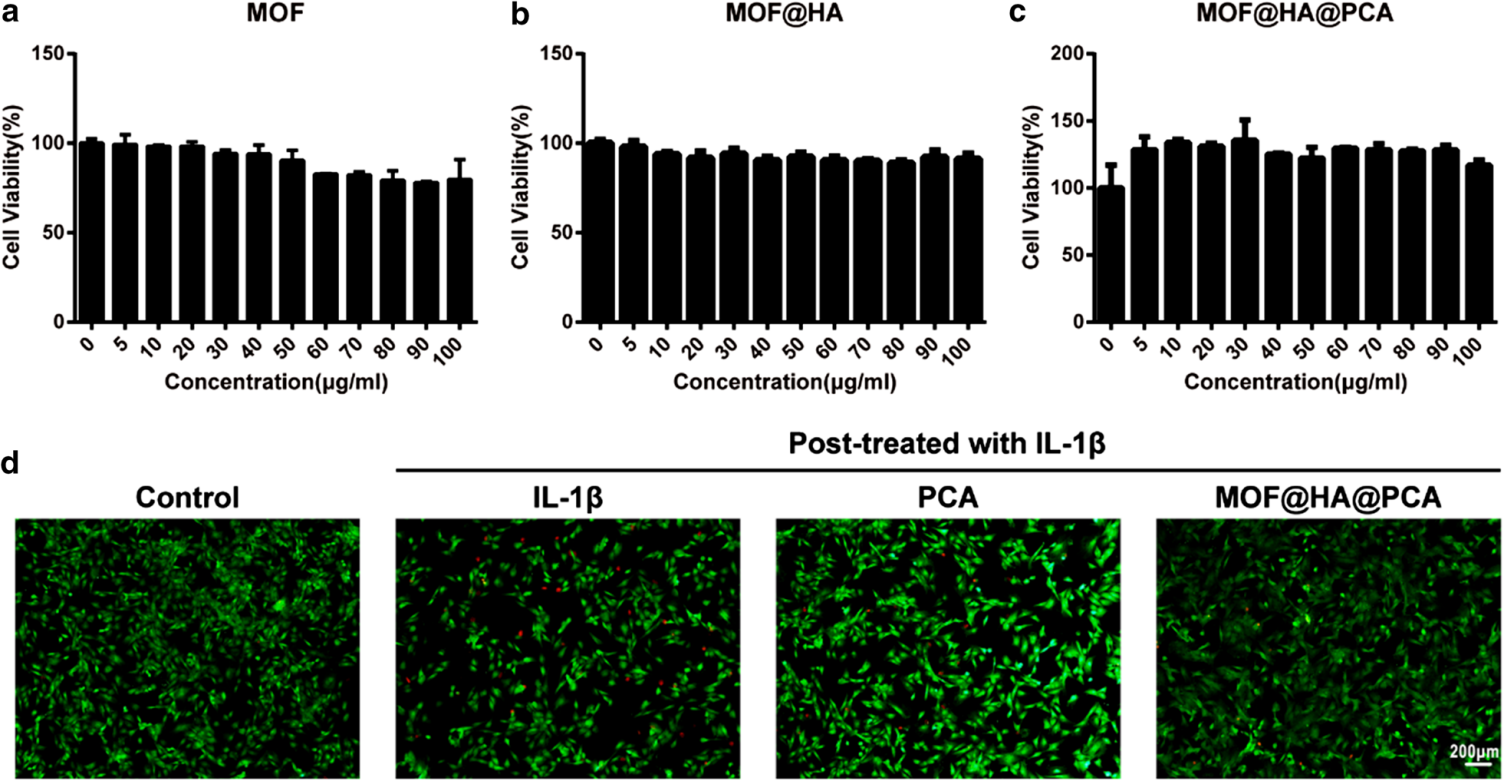
**a**–**c** Cytotoxicity of MOF, MOF@HA and MOF@HA@PCA in chondrocytes. **d** Cell viability was determined by live/dead assay of IL-1β-induced chondrocytes after treating with PCA or MOF@HA@PCA for 24 h (scale bar: 200 μm)

Cell viability was determined to evaluate the proliferation ability of NPs on chondrocytes by using MTT assay protocol. MOF and MOF@HA NPs were found to be biocompatible and non-toxic range from 0 to100 µg/mL, as the cell viability was found to be more than 77% and 85%, respectively, after 24 h of incubation (Fig. 2a, b). After PCA loading, MOF@HA@PCA promoted cell proliferation in the concentration range of 0 to 100 μg/mL, and showed the highest cell viability at concentration of 30 μg/mL (Fig. 2c). Thus, 30 μg/mL MOF@HA@PCA was used for subsequent experiments.

The live/dead assay was used to evaluate cell viability. The results showed that a comparatively high percentage of apoptotic cells was observed in the OA group, higher than that in the treatment groups (Fig. 2d). However, the proportion of live cells/dead cells in the MOF@HA@PCA group was rather high, similar to that in the control group, indicating that MOF@HA@PCA cultivation had not led to chondrocytes apoptosis in vitro and showed higher cell viability than that without treatment.

### ROS production induced by MOF

ROS generated by MOF was evaluated by the fluorescence of DCF. After the cells were incubated with Rosup, strong green fluorescence was observed in the cytoplasm (the fluorescence rate was 34.83%), indicating that the level of ROS in the cells increased. However, Additional file 1. Fig. S7 showed that the MOF treatment group elicited significantly weak positive staining (green) (7.13%) by DCFH, similar to that of control group (7.47%), indicating that the level of ROS induced by MOF was extremely low after incubation in medium for 24 h.

### Anti-inflammatory effects on IL-1β-induced chondrocytes

**Fig. 3:**
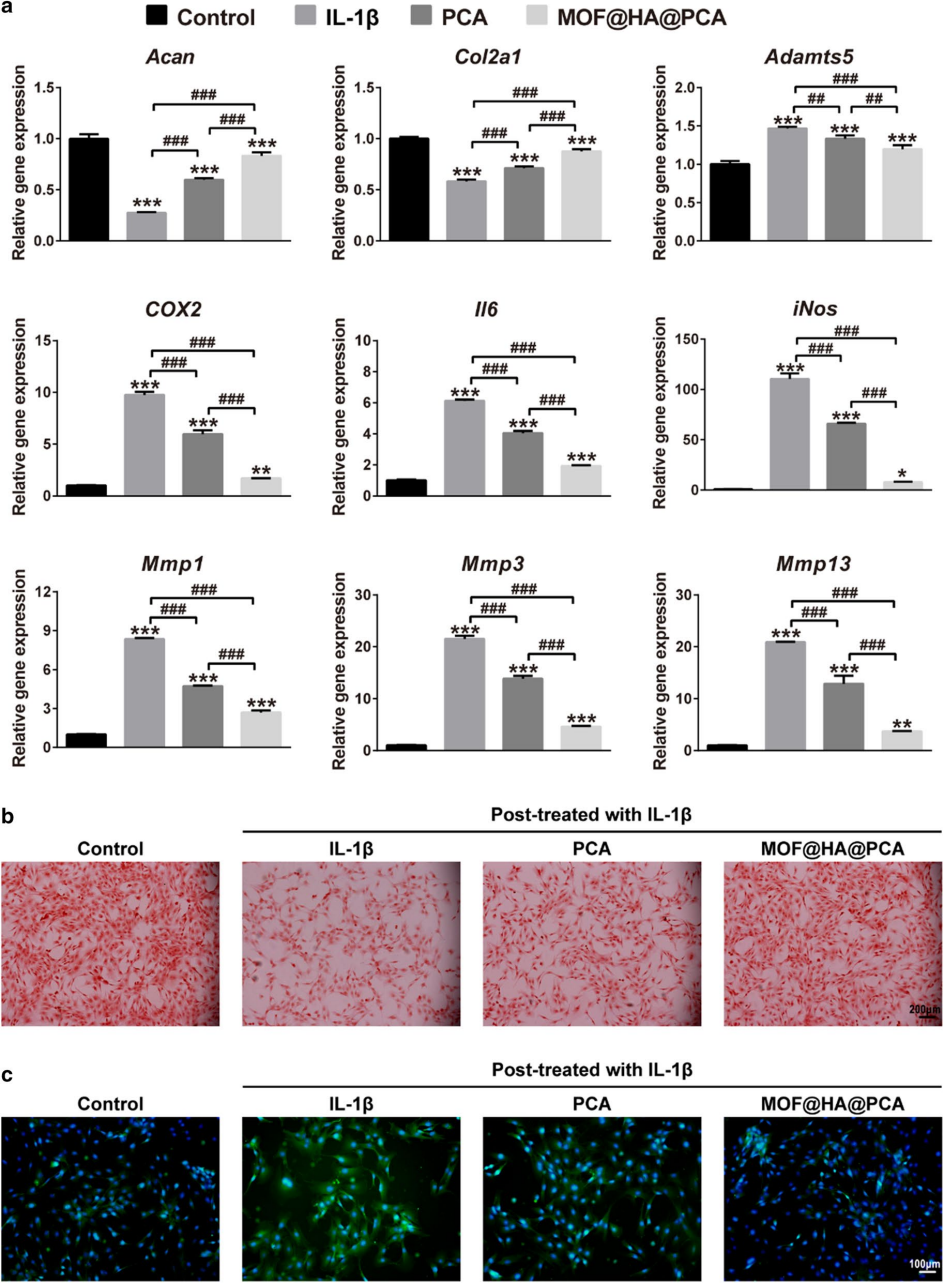
**a** Relative mRNA levels of chondrogenic markers *(Acan, Col2a1*) and OA-relative genes (*Adamts5, COX2, Il6, iNos, Mmp1, Mmp3, Mmp13*). Data was presented as the mean ± SD (n = 3). *, ^*#*^p < 0.05; **, ^##^*p* < 0.01; ***, ^###^*p* < 0.001. **b** Safranin O stained for GAG production (scale bar: 200 µm). **c** The expression of MMP-13 was detected by immunofluorescent staining (scale bar: 100 µm)

The effect of MOF@HA@PCA on the expression of cartilage-specific markers (including *Acan* and *Col2a1*) and OA-related catabolic markers (including *Adamts5, COX2, Il6, iNos, Mmp1, Mmp3, Mmp13*) was analyzed by qRT-PCR (Fig. 3a). After IL-1β stimulation, the expression of the cartilage-specific markers in OA group was the lowest in all groups, with a remarkable decrease of ~ 72.51% (for *Acan*), 41.74% (for *Col2a1*) compared with the control group. However, pretreatment with PCA and MOF@HA@PCA, the expression of *Acan* and *Col2a1* was significantly up-regulated by 2.17-fold and 3.03-fold (for *Acan*), 1.22-fold and 1.51-fold (for *Col2a1*), respectively, compared with OA group. In contrary, as showed in Fig. 3a, the expression of OA-related gens in OA group was sharp up-regulated compared with all groups, whereas slightly down-regulated in treatment groups. In particular, the data showed that MOF@HA@PCA significance down-regulated the expression of *Adamts5, COX2, Il6, iNos, Mmp1, Mmp3, Mmp13* by 18.53%, 82.69%, 68.64%, 92.96%, 67.72%, 78.63%, and 82.28% compared with IL-1β treatment group after treatment for 24 h (Fig. 3a).

The Safranin O staining showed that the IL-1β treatment groups caused weaker positive staining (red) by safranin O compared with normal group, indicating that GAG secretion decreased rapidly in OA environment (Fig. 3b). However, GAG was the most abundant and homogeneously distributed around the cells in the MOF@HA@PCA group after 24 h of cultivation, which was consistent with the results of qRT-PCR (Fig. 3a, b).

The expression of OA biomarkers was evaluated by immunofluorescence staining to further prove OA therapeutic effect of NPs, which showed the strong positive staining of MMP-13 in OA, PCA and MOF@HA@PCA groups, particularly in only IL-1β stimulation group (Fig. 3c). The MMP-13, which is the key marker of OA-related pathological environment and indicators of inflammatory catabolism, was less positively secretion in the MOF@HA@PCA group than in other IL-1β treatment groups, indicating that it may effectively suppress the inflammatory reaction induced by IL-1β in vitro.

All these results suggested that MOF@HA@PCA could inhibited the degradation of collagen type II to protect chondrocytes, and pronounced the effect of anti-inflammatory in the OA-related pathological microenvironment.

### MOF@HA@PCA promote cartilage regeneration in vivo

**Fig. 4:**
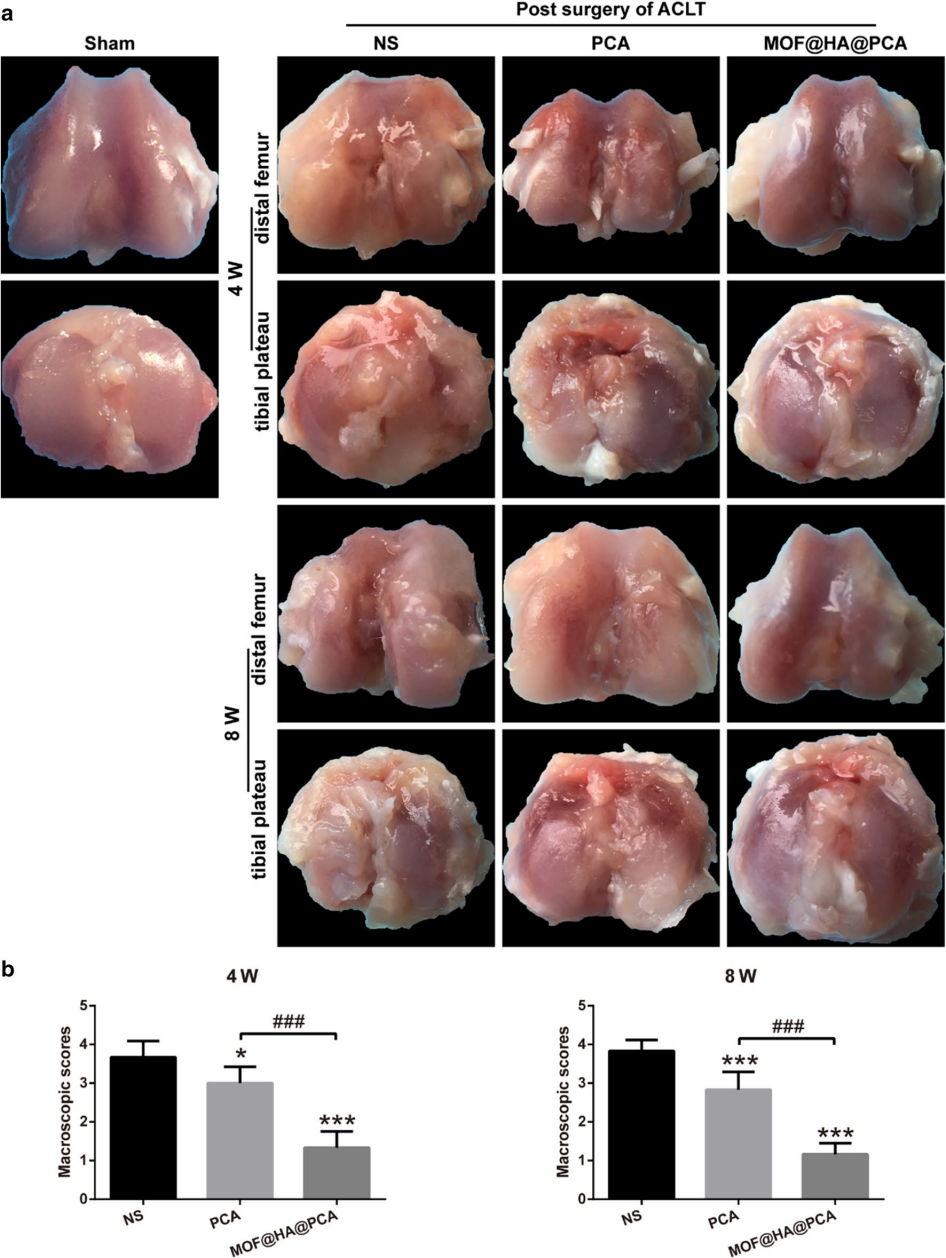
**a** Macroscopic appearance and **b** Macroscopic scores of distal femur and tibial plateau from rats after treatment for 4 and 8 weeks. Data was presented as the mean ± SD (n = 6). *, ^#^*p* < 0.05; **, ^*##*^*p* < 0.01; ***, ^###^*p* < 0.001

**Fig. 5:**
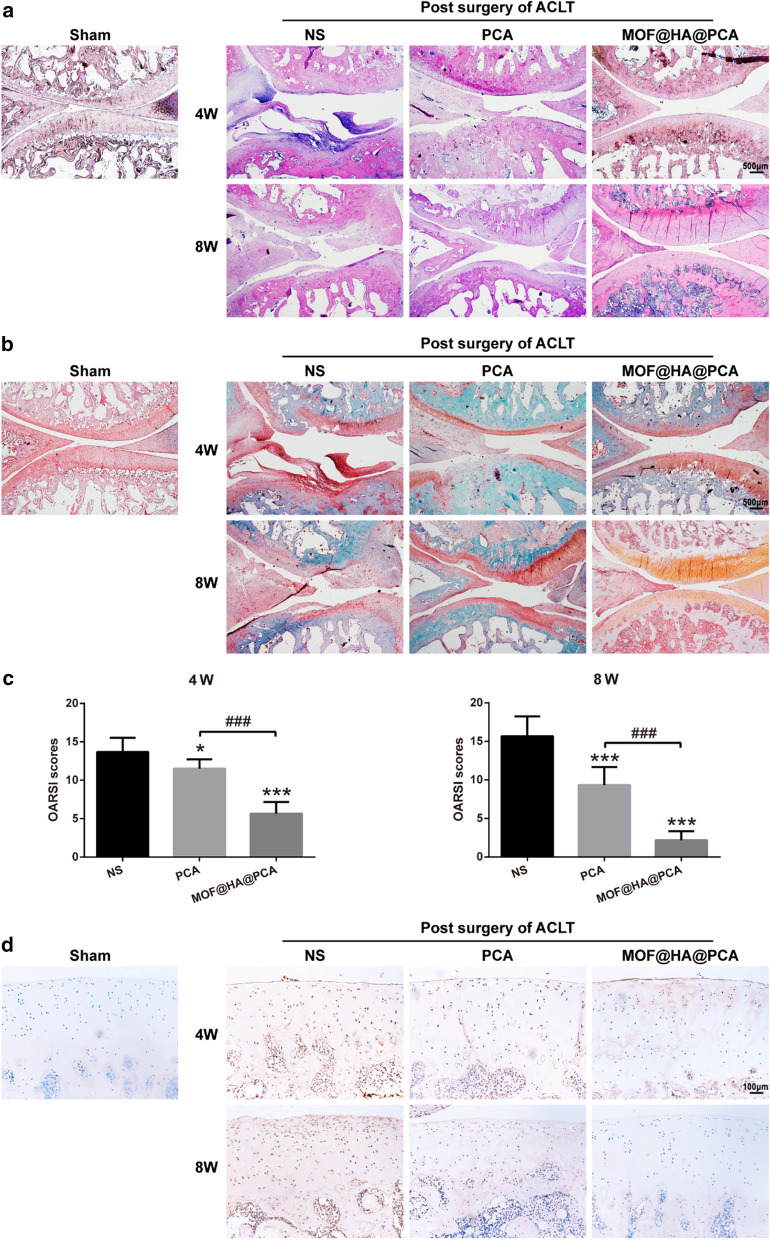
**a** Hematoxylin and eosin (HE) and **b** safranin O/fast green staining of cartilage after treatment for 4 and 8 weeks (scale bar: 500 μm). **c** OARSI scores for histology of articular cartilage after treatment for 4 and 8 weeks. Data were presented as the mean ± SD (n = 6). *, ^#^*p* < 0.05; **, ^##^*p* < 0.01; ***, ^*###*^*p* < 0.001. **d** Immunohistochemical staining of MMP-13 in cartilage after treatment for 4 and 8 weeks (scale bar: 100 μm)

To further prove the repairing effect of MOF@HA@PCA on degenerative cartilage, the joints were collected for macroscopic evaluation and histologic assessments at 4 and 8 weeks after treatment (Figs. 4 and 5). There was no obvious macroscopic cartilage abrasion in sham group, while OA characteristics, including cartilage surface erosion and osteophyte formation, were observed in all ACLT groups after 4 and 8 weeks of treatment (Fig. 4a). Especially in the NS group, a large area of cartilage erosion and massive osteophyte formation was exhibited. After therapy for 8 weeks, the destructions in cartilage were still observed in NS and PCA groups, whereas defects in MOF@HA@PCA group obviously formed glossy and smooth cartilage-like tissues with a score significantly reduction of 67.57% and 71.05% compare with NS group after 4 and 8 weeks of treatment, respectively (Fig. 4a, b).

In addition, the histological morphological findings were showed by H&E staining, which was consistent with that in macroscopic assessment (Fig. 5a). The superficial fibrillation and progressive cartilage degenerations were shown in NS group, and minor surface destabilization, thinner cartilage as well as matrix vertical fissures were observed in PCA group. Notably, only minor superficial fibrillations were exhibited in the periphery of cartilage surface in MOF@HA@PCA group, suggesting huge repair capability to attenuate the progression of OA.

The safranin O/fast green staining, assessing the large changes in content of proteoglycan in cartilage matrix, was shown in Fig. 5b. The NS group showed large areas of cartilage defect, with proteoglycan loss and surface erosion. But it could be seen that the content of proteoglycan in the cartilage matrix of the PCA and MOF@HA@PCA group was lesser loss and relatively distributed with strong positive staining (red) of Safranin. In comparison with NS group, MOF@HA@PCA-treated rats demonstrated distinct anti-inflammatory effects and cartilage regeneration with minimum OARSI score reduction of 60.28% and 87.26% after 4 and 8 weeks of therapy, respectively (Fig. 5c).

The expression of MMP-13 was analyzed by immunohistological staining to reflect the damage degree of cartilage caused by OA in vivo (Fig. 5d). In the superficial and middle zones of cartilage, intense positive staining of the damage chondrocytes was shown in the NS group, while the dark brown rarely seen in the control group. In contrast, the secretion of MMP-13 was weakened after treatment with PCA and MOF@HA@PCA. In particular, almost negative staining of MMP-13 was shown in MOF@HA@PCA group joints, suggesting it greatly reducing the level of inflammation in the OA cartilage.

These data indicated that MOF@HA@PCA significantly attenuate the progression of OA and accelerated cartilage regeneration in vivo.

## Discussion

In this study, we first used HA to modify pH-responsive nanocarrier MOF, and subsequently loaded anti-inflammatory drug PCA to form MOF@HA@PCA for therapy of OA. The MOF-based NPs act as drug carries due to their properties of controlled drug release and good biocompatibility. And with the function of HA and PCA, MOF@HA@PCA could reduce inflammation of chondrocytes in vitro and prevent the progress of OA in vivo to protect cartilage tissues.

The microenvironment of inflamed-affect joint is acidity, and thus may be a vital stimulus of triggered drug release systems for precise therapy of OA [54, 55]. In our study, we found that the smaller release amount of PCA from MOF@HA@PCA was observed at pH 7.4, and the release amount increased with the reduction of pH value (pH 5.6) (Fig. 1f), indicating that due to the pH-triggered release property of MOF, the PCA was sustained released on-demanded according to the severity of inflammation in joint. This finding is consistent with previous studies that the accelerated release of PCA can be importantly attributed to the degradation of Fe^3+^ ions in an acidic condition, making it suitable for MIL-100(Fe) to form pH-guided drug delivery systems [56, 57]. Simultaneously, the pH-responsive degradation of MIL-100(Fe) would prolong the release of PCA through drug encapsulation within MOFs, resulting in continuous therapy effects.

Further, adding HA, a natural biodegradable and biocompatible biopolymer [58], could improve the stability and dispersibility of MOF NPs (Fig. 1a–c). The in vitro and in vivo study (Fig. 3, 4 and 5) supported the idea that HA could suppress the production of the proinflammatory cytokines and enhance lubrication on the cartilage surface to prevent the progression of OA. The result is in accordance with those who reported that HA could effectively suppress IL-1β–stimulated production of matrix metalloproteinases (MMPs) and free-radicals to protect chondrocytesdue to the binding of HA to CD44 on chondrocytes [59, 60]. It is worth noting that HA can inhibit IL-1β-induced chondrocyte apoptosis and promote chondrocytes survival [61, 62], which is consistent with our present study (Fig. 2). HA coating physically protects the chondrocytes from inflammatory factors and degrading enzymes that are frequently found in an OA microenvironment. Thus, MOF@HA NPs may attract more attention as drug carriers due to its low cytotoxicity, stability and biocompatibility under physiological conditions [63].

It has been reported that PCA is a potential anti-inflammatory agent by inhibiting the expression of iNOS and cyclooxygenase-2 (COX2) [64, 65]. And PCA can also maintain cell integrity and prevent the elevation of inflammatory markers during the cell proliferation [40]. In our studies, we observed that after loading of PCA, MOF@HA@PCA could suppress the IL-1β-stimulated inflammatory mediators (iNOS and COX2), the major proteolytic enzymes (matrix metalloproteinases, MMPs and disintegrins and metalloproteinase with thrombospondin motifs, ADAMTSs) and pro-inflammatory cytokines (interleukin-6, IL-6) to alleviate the progress of OA and degradation of cartilage in vitro (Fig. 3a, c). Additionally, PCA was able to promote cell proliferation and glycosaminoglycan (GAG) production of chondrocytes [66], consistent with the up-regulation of *Col2a1* and *Acan* **(**Figs. 3a, b and 5b**)**. These data also support that IA injection of MOF@HA@PCA remarkably reduced the level of inflammatory cytokines and alleviated the cartilage lesions compared with other treatment groups after 4 and 8 weeks of treatment (Figs. 4 and 5). Thus, MOF@HA@PCA could be effectively therapy of OA after IA injection.

## Conclusion

In summary, we successfully constructed MOF@HA@PCA NPs by modifying MOF NPs with HA and subsequently loading with PCA molecules to achieve pH-sensitive system for therapy of OA. The novel nanoparticle-based system MOF@HA@PCA could prolong the residence time of the PCA in the joint cavity and improve the therapeutic effect of OA. In response to the acidic environment of OA in joints, MOF@HA@PCA could titrate drug release according to the degree of disease and effectively attenuate OA progression. Therefore, this system provides a useful strategy for therapy of OA in clinical applications.

## Supplementary information

**Additional file 1:** **Figure S1. **The chemical structures of HA (hyaluronic acid). **Figure S2. **The chemical structures of PCA (protocatechuic acid). **Figure S3.** The chemical structures of MOF. **Figure S4.** The typical chromatogram of PCA. **Figure S5. **The quantification of Fe^3+^ by using o-phenanthroline. **Figure S6. **Degradation study of MOF at different pH. **Figure. S7** ROS production induced by MOF after incubation in chondrocytes for 24 h.

## Data Availability

The datasets used and/or analyzed during the current study are available from the corresponding author on reasonable request.
