# Supplementary material for: pH-responsive and hyaluronic acid-functionalized metal–organic frameworks for therapy of osteoarthritis
Source: J Nanobiotechnology. 2020 Sep 29;18:139. doi: 10.1186/s12951-020-00694-3 (PMC7523381; doi:10.1186/s12951-020-00694-3)
Supplement: Supplementary file 1 — Additional file 1: Figure S1. The chemical structures of HA (hyaluronic acid). Figure S2. The chemical structures of PCA (protocatechuic acid). Figure S3. The chemical structures of MOF. Figure S4. The typical chromatogram of PCA. Figure S5. The quantification of Fe3+ by using o-phenanthroline. Figure S6. Degradation study of MOF at different pH. Figure. S7 ROS production induced by MOF after incubation in chondrocytes for 24 h. [file 12951_2020_694_MOESM1_ESM.pdf]

pH-responsive and hyaluronic acid-functionalized metal-organic frameworks for therapy of osteoarthritis

Feng Xiong[1,2,3,4], Zainen Qin[1,2,5], Haimin Chen[1,2,5], Qiumei Lan[1,3,5], Zetao Wang[1], Nihan Lan[1,2,5,*], Yuan Yang[1,2,4,*], Li Zheng[1,2,*], Jinmin Zhao[1,2,3,6], Dan Kai[7]

1 Guangxi Engineering Center in Biomedical Material for Tissue and Organ Regeneration, The First Affiliated Hospital of Guangxi Medical University, Nanning, 530021, China

2 Guangxi Collaborative Innovation Center for Biomedicine, The First Affiliated Hospital of Guangxi Medical University, Nanning, 530021, China

3 Department of Orthopaedics Trauma and Hand Surgery, The First Affiliated Hospital of Guangxi Medical University, Nanning, 530021, China

4 Orthopaedics, Langdong Hospital of Guangxi Medical University, The First Affiliated Hospital of Guangxi Medical University, Nanning, 530021, China

5 Life Sciences Institute, Guangxi Medical University, Nanning, 530021, China

6 Guangxi Key Laboratory of Regenerative Medicine, Life Sciences Institute, The First Affiliated Hospital of Guangxi Medical University, Nanning, 530021, China

7 Institute of Materials Research and Engineering (IMRE), A*STAR, 2 Fusionopolis Way, #08-03 Innovis, 138634, Singapore

* Co-Corresponding authors: Nihan Lan, Yuan Yang, Li Zheng

E-mail addresses: 1142963164@qq.com (Prof. Nihan Lan), yangy062@sina.com (Prof. Yuan Yang), and zhengli224@163.com, Fax: +86-07715306456 (Prof. Li Zheng)

[1] Feng Xiong, Zainen Qin, Haimin Chen contributed as first authors.

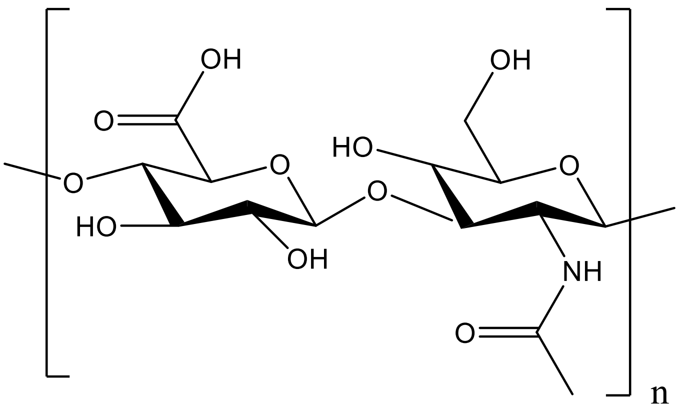

Figure S1. The chemical structures of HA (hyaluronic acid) [1, 2].

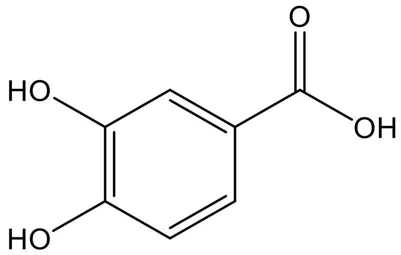

Figure S2. The chemical structures of PCA (protocatechuic acid) [3].

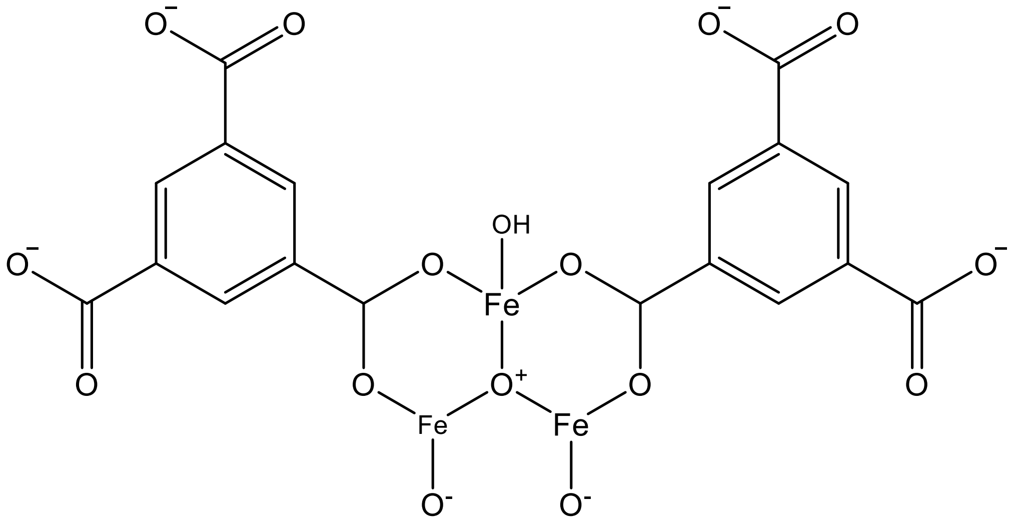

Figure S3. The chemical structures of MOF [4].

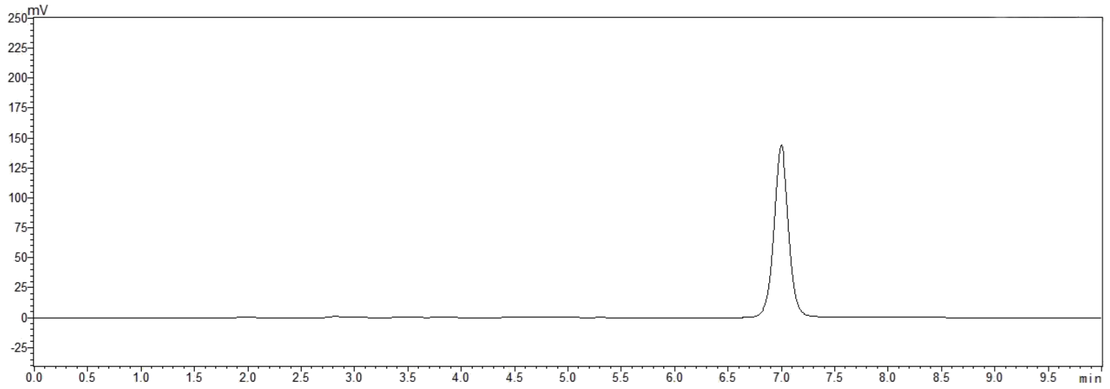

Figure S4. The typical chromatogram of PCA.

1. Quantify the amount of HA on MOF NPs

The MOF@HA NPs could be obtained by mixing MOF NPs with HA (Sigma-Aldrich, USA) at weight ratio of 2:1 for 24 h. The mixtures were washed with water for three times to remove the free HA, then MOF@HA NPs were obtained after lyophilization under vacuum. We quantified the amount of HA on MOF NPs and calculated the rate of HA on MOF NPs (HA, %) by the following formula:

$$\text{HA (\%)} = \frac{\text{weight of MOF@HA - weight of MOF feeding}}{\text{weight of MOF feeding}} \times 100\%$$

2. Quantitative measurement of released Fe^{3+} by the method of o-phenanthroline [5]

The degradation of MOF was investigated by quantitative measurement of released Fe^{3+} at different pH (**Figure S5 & 6**). $FeCl_3$ (4 mM) was diluted to different concentration and reduced to Fe^{2+} by Vitamin C (10 mM, 1 mL) for 5 min, then the mixture was reacted with o-phenanthroline (0.1%, 1 mL) and the red complex was produced, which absorbance is at 510 nm. The volume of the reaction system was 5 mL. The absorption spectra of different samples were obtained using an UV-vis spectrometer. The absorbance intensity at 510 nm was correlated with the Fe^{3+} in concentration. The

obtain standard curve is y=0.00548x+0.04226 (y: absorbance value at 510 nm; x: concentration of Fe^{3+}, R^2= 0.9998) (**Fig. S5**).

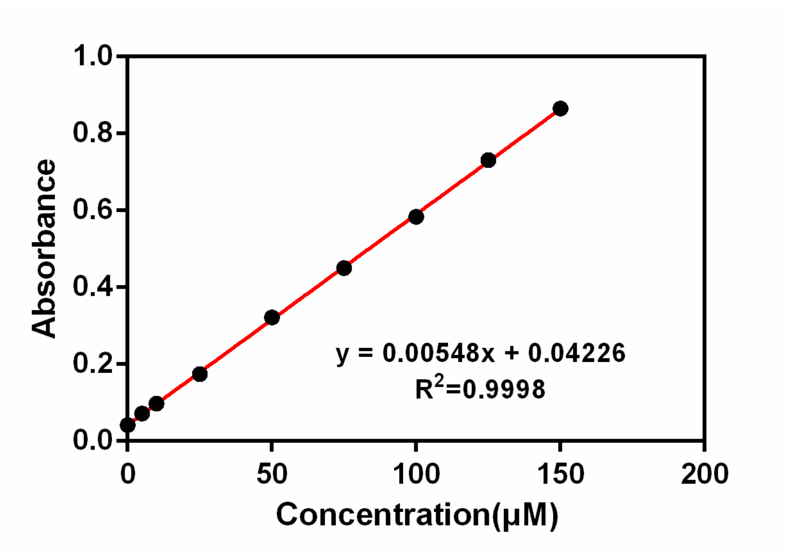

Figure S5. The quantification of Fe^{3+} by using o-phenanthroline. The absorption spectra of inset: The standard curve for absorbance value at 510 nm. y=0.00548x+0.04226, R^2=0.9998.

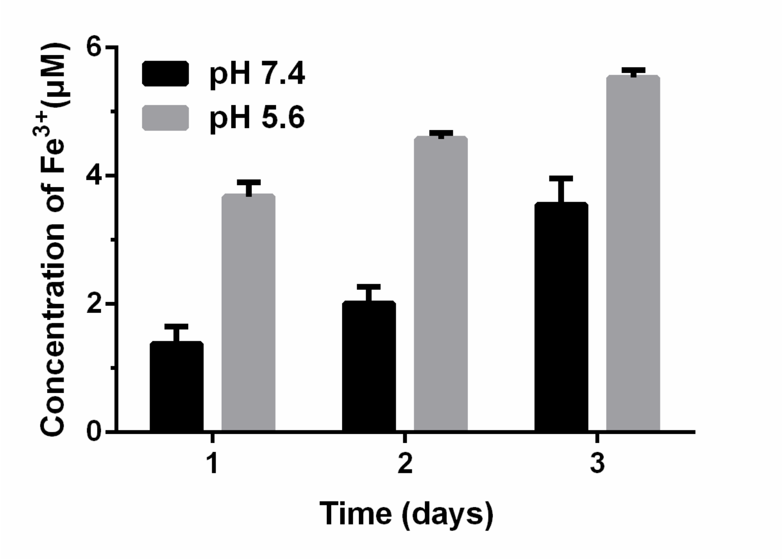

Figure S6. Degradation study of MOF at different pH. The quantitative analysis of the Fe^{3+} released from MOF in various pH environment. The data represents the mean ± SD (n=3).

3. ROS production from MOF in chondrocytes

Reactive Oxygen Species Assay Kit (Beyotime Biotechnology, China) was used to detect the ROS generation of intracellular induced by MOF [6, 7].

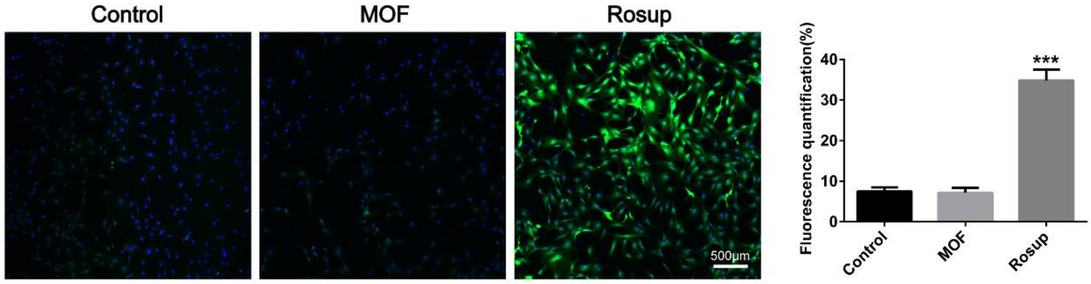

Figure. S7 ROS production induced by MOF after incubation in chondrocytes for 24h. Data was presented as the mean ± SD (n=3). * $p < 0.05$; ** $p < 0.01$; *** $p < 0.001$.

[1] Burdick JA, Prestwich GD. Hyaluronic acid hydrogels for biomedical applications. Advanced materials. 2011;23(12):H41-56.

[2] Highley CB, Prestwich GD, Burdick JA. Recent advances in hyaluronic acid hydrogels for biomedical applications. Current opinion in biotechnology. 2016;40:35-40.

[3] Krzysztoforska K, Mirowska-Guzel D, Widy-Tyszkiewicz E. Pharmacological effects of protocatechuic acid and its therapeutic potential in neurodegenerative diseases: Review on the basis of in vitro and in vivo studies in rodents and humans. Nutritional neuroscience. 2019;22(2):72-82.

[4] Tahereh AV, Reza NJM, Leila P. (Fe)MIL-100-Met@alginate: a hybrid polymer–MOF for enhancement of metformin's bioavailability and pH-controlled release. New Journal of Chemistry. 2018:10.1039.

[5] Wang D, Zhou J, Chen R, Shi R, Xia G, Zhou S, et al. Magnetically guided delivery of DHA and Fe ions for enhanced cancer therapy based on pH-responsive degradation of DHA-loaded Fe3O4@C@MIL-100(Fe) nanoparticles. Biomaterials. 2016;107:88-101.

[6] Sun L, Chen L, Bai L, Xia Y, Yang X, Jiang W, et al. Reactive oxygen species mediates 50-Hz magnetic field-induced EGF receptor clustering via acid sphingomyelinase activation. International journal of radiation biology. 2018;94:678-684.

[7] Deng S, Dai G, Chen S, Nie Z, Zhou J, Fang H, et al. Dexamethasone induces osteoblast apoptosis through ROS-PI3K/AKT/GSK3beta signaling pathway. Biomed Pharmacother. 2019;110:602-608.
